# Large-scale production of tauroursodeoxycholic acid products through fermentation optimization of engineered *Escherichia coli* cell factory

**DOI:** 10.1186/s12934-019-1076-2

**Published:** 2019-02-08

**Authors:** Yingpeng Xu, Li Yang, Shujuan Zhao, Zhengtao Wang

**Affiliations:** The SATCM Key Laboratory for New Resources & Quality Evaluation of Chinese Medicine, The MOE Key Laboratory for Standardization of Chinese Medicines and Shanghai Key Laboratory of Compound Chinese Medicines, Institute of Chinese Materia Medica, Shanghai University of Traditional Chinese Medicine, Shanghai, 201203 People’s Republic of China

**Keywords:** TUDCA, Fermentation, Biotransformation, *Escherichia coli*, Process optimization, Deep-tank static process

## Abstract

**Background:**

Bear bile powder is a valuable medicinal material characterized by high content of tauroursodeoxycholic acid (TUDCA) at a certain ratio to taurochenodeoxycholic acid (TCDCA). We had created an engineered *E. coli* harboring two-step bidirectional oxidative and reductive enzyme-catalyzing pathway that could rapidly convert TCDCA to TUDCA at a specific percentage in shake flasks.

**Results:**

We reported here the large-scale production of TUDCA containing products by balancing the bidirectional reactions through optimizing fermentation process of the engineered *E. coli* in fermenters. The fermentation medium was firstly optimized based on M9 medium using response surface methodology, leading to a glycerol and yeast extract modified M9-GY medium benefits for both cell growth and product conversion efficiency. Then isopropylthio-β-galactoside induction and fed-stock stage was successively optimized. Finally, a special deep-tank static process was developed to promote the conversion from TCDCA to TUDCA. Applying the optimal condition, fermentation was performed by separately supplementing 30 g refined chicken bile powder and 35 g crude chicken bile powder as substrates, resulting in 29.35 ± 2.83 g and 30.78 ± 3.04 g powder products containing 35.85 ± 3.85% and 27.14 ± 4.23% of TUDCA at a ratio of 1.49 ± 0.14 and 1.55 ± 0.19 to TCDCA, respectively, after purification and evaporation of the fermentation broth. The recovery yield was 92.84 ± 4.21% and 91.83 ± 2.56%, respectively.

**Conclusion:**

This study provided a practical and environment friendly industrialized process for producing artificial substitute of bear bile powder from cheap and readily available chicken bile powder using engineered *E. coli* microbial cell factory. It also put forward an interesting deep-tank static process to promote the enzyme-catalyzing reactions toward target compounds in synthetic biology-based fermentation.

**Electronic supplementary material:**

The online version of this article (10.1186/s12934-019-1076-2) contains supplementary material, which is available to authorized users.

## Background

Synthetic biology is a powerful strategy for producing valuable natural products from inexpensive starting materials. The basic work of this strategy is to integrate a heterologous pathway in a microbial host cell where balancing the multiple enzyme-catalyzed reactions of the recreated pathway is essential for high production of committed products. A number of technologies have been developed to balance the metabolic flux including structure gene selection and codon optimization [1], gene transcriptional level controlled by varied strength promoters or gene dosage [1, 2], enzyme co-location through fusion protein or co-expressing with synthetic protein scaffolds [3, 4], and microbial co-culture consortium [5, 6]. Fermentation optimization also play important role in promoting the titer, yield and productivity of target compounds [2].

Bear bile powder is precious traditional medicinal material. It has been used as anti-convulsant drugs for curing hepatic and biliary disorders and other diseases in Asia area for thousands of years [7–9]. Modern pharmacological studies revealed it has a wide range of activities such as anti-inflammatory [10], anti-liver fibrosis [11], neuroprotective [12], and antioxidant [13], which made its application widely spread around the world [8]. Bear bile powder is the dried gallbladder bile collected from the endangered Ursidae species like black bear (*Selenarctos thibetanus*) and brown bear (*Ursus arctos*) [7, 8]. Currently, bear bile powder for medicinal usage is predominantly from the drainage bear bile extracted from farmed bears with “Free-dripping Fistula Technique” by implanting a duct or making an artificial fistula in the liver of the bears, which is extremely inhumane and would kill them due to chronic infections or liver cancer [8]. The high value of bear bile but the cruel collection method prompted researchers to find alternatives based on its therapeutic effects and the specific chemical characteristics [8].

Bile acids, a group of steroids with C-17 side chains, are the main bioactive ingredients of bear bile especially ursodesoxycholic acid (UDCA) or its physiologically active form tauroursodesoxycholic acid (TUDCA) and taurochenodeoxycholic acid (TCDCA). The high content of UDCA or TUDCA is generally considered as the special profile of bear bile and used to distinguish it from other animal bile [7, 14]. The ratio of TUDCA to TCDCA ranged from 0.82 to 1.76 (average 1.1 ± 0.3) in eight batches of commercial bear bile powder samples [14], which could be regarded as the distinct character of bear bile. The authentication and standard quality work from 20 batches of drainage bear bile powder samples in our lab revealed that the average content of TUDCA was 26.5%, the ratio of TUDCA to TCDCA was from 1.0 to 1.5 and the content of TUDCA and TCDCA was from 41 to 59% [15].

Till now, UDCA and TUDCA synthesized through chemical synthesis are the only acceptable substitutes of bear bile [16–18], from the pharmacological point of view. Biotransformation using whole-cell biocatalysts or chemo-enzymes have also attracted researchers for its mild, steerable and environment-friendly properties [19, 20]. Both chemical synthesis and current biotransformation approaches required pure and rare compounds as precursors, substrates, or cofactors [16–20], thus limited in industrial application.

**Fig. 1:**
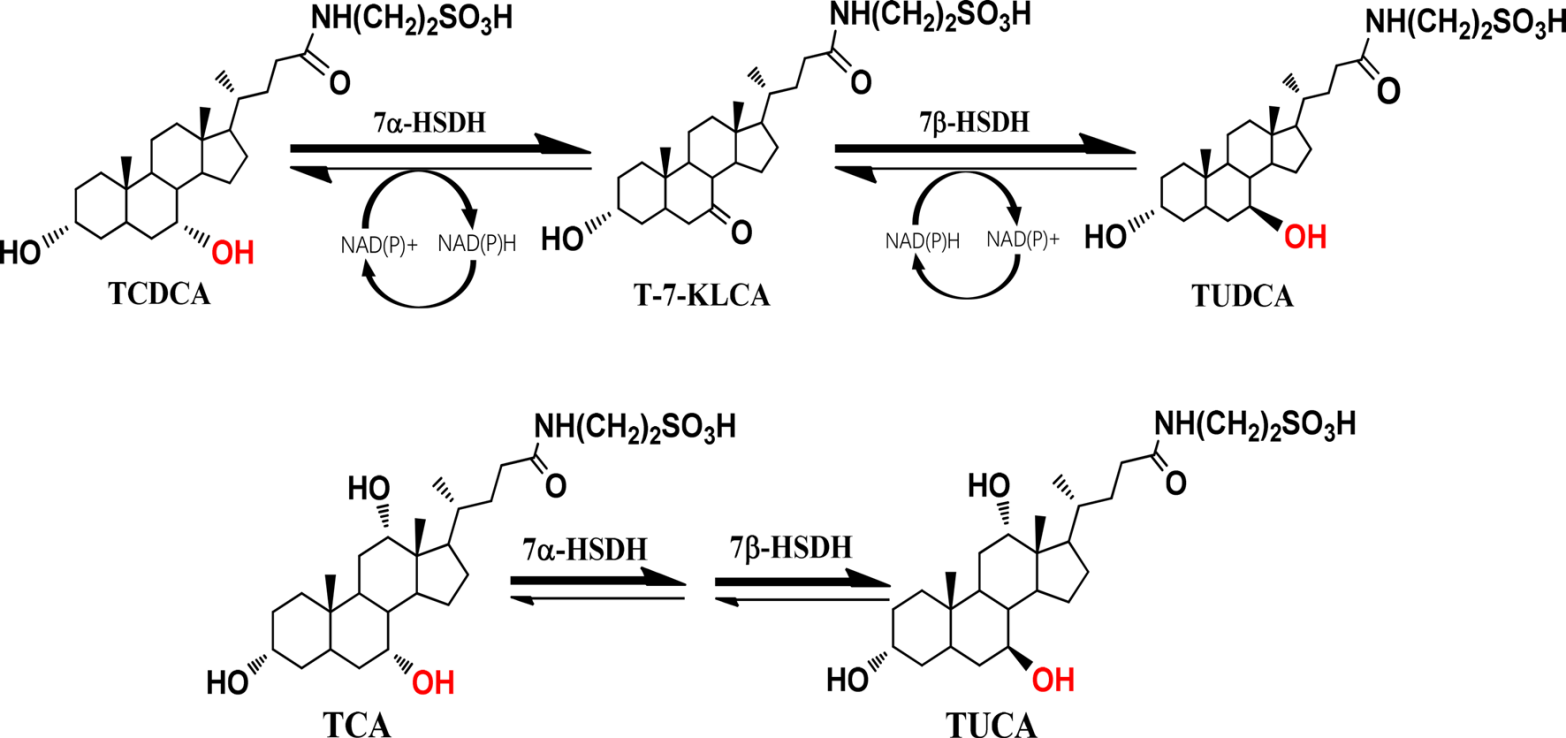
Reaction catalyzed by 7α-HSDH and 7β-HSDH

Bacterial 7α-hydroxysteroid dehydrogenase (7α-HSDH) and 7β-hydroxysteroid dehydrogenase (7β-HSDH) have both oxidative and reductive activities and can interconvert TCDCA and TUDCA coupling with NADPH or NADH as co-factors [20, 21] (Fig. 1). Utilizing the property of 7α-HSDH and 7β-HSDH, we had created an engineered *Escherichia coli* with 7α-HSDH and 7β-HSDH genes which could convert a certain proportion of TCDCA to TUDCA via tauro-7-keto lithocholic acid (T-7-KLCA) intermediate using chicken bile powder as substrates [22]. The oxidative and reductive activities of 7α-HSDH and 7β-HSDH are affected by temperature, available oxygen, pH value of the media, and the fermentation time in shake flasks [22–24]. Here, in order to make the engineered *E. coli* work feasible for industrial application, we performed process optimization to balance the bidirectional reactions converting TCDCA to TUDCA and to improve the production as well. Medium composition was optimized with response surface methodology (RSM), a useful tool reducing the number of experiments without neglecting the interactions among the parameters [25, 26], and a special deep-tank static process was developed.

Employing the optimized condition, we carried out fermentation in stirred-tank fermenter using refined chicken powder and crude chicken bile powder as substrates and prepared the fermentation broth into powder products consisting of nearly equal content of TUDCA to natural bear bile acid powder. To our knowledge, this is the first report applying engineered whole-cell factory to make products in large scale containing TUDCA at a certain ratio to TCDCA as in the rare bear bile powder medicinal material.

## Results

### Evaluation of experimental design of Box–Behnken Design of RSM

The results of 29 runs from Box–Behnken Design (BBD) experiments to study the effects of the four independent factors, glucose (factor A), glycerol (factor B), tryptone (factor C), and yeast extract (factor D), on the cell density (OD_600_) and conversion efficiency of TUDCA were loaded into Design-Expert 8.0.6.1 software for regression analysis (Table 1). The maximum experimental value of OD_600_ was 13.29 and 22.96% for conversion efficiency based on RSM. The regression analysis data were fitted to a quadratic model and the second order regression equations obtained on OD_600_ (1) and conversion efficiency (2) were actual models as following:

1 $$\begin{aligned} {\text{Y}} = &10.59 + 2.23 * {\text{A}} - 0.21 * {\text{B}} + 0.48 * {\text{C}} + 1.43 * {\text{D}} - 0.23 * {\text{A}}* {\text{B}} \\ & + 1.17 * {\text{A}}*{\text{C}} + 0.66 * {\text{A}}*{\text{D}} + 0.58 * {\text{B}}*{\text{C}} + 0.22 * {\text{B}}*{\text{D}} - 0.52 * {\text{C}}*{\text{D}} \\ & - 1.52 * {\text{A}}*{\text{A}} + 0.42 * {\text{B}}*{\text{B}} - 0.18 * {\text{C}}*{\text{C}} - 1.23 * {\text{D}}*{\text{D }} \\ \end{aligned}$$

**Table 1:** Experimental designs and the results of the Box–Behnken Design

Std	Run	Factors				Responses	
		A-glucose (g/L)	B-glycerol (mL/L)	C-tryptone (g/L)	D-yeast extract (g/L)	OD_600_	Conversion efficiency (%)
22	1	10	30	10	0	8.36	1.41
14	2	10	30	0	10	10.25	0.17
9	3	0	15	10	0	4.37	12.54
5	4	10	15	0	0	5.64	1.37
23	5	10	0	10	20	10.91	1.11
8	6	10	15	20	20	10.91	3.26
2	7	20	0	10	10	12.04	0.19
28	8	10	15	10	10	11.44	0.00
4	9	20	30	10	10	10.43	0.03
13	10	10	0	0	10	10.81	0.58
29	11	10	15	10	10	12.50	0.00
26	12	10	15	10	10	9.74	0.00
11	13	0	15	10	20	6.25	*22.96*
12	14	20	15	10	20	*13.29*	0.45
1	15	0	0	10	10	7.28	3.13
21	16	10	0	10	0	9.51	0.03
25	17	10	15	10	10	8.65	0.01
16	18	10	30	20	10	12.66	0.31
3	19	0	30	10	10	6.61	13.42
27	20	10	15	10	10	10.62	0.00
10	21	20	15	10	0	8.76	0.02
17	22	0	15	0	10	8.50	20.25
19	23	0	15	20	10	6.05	13.58
18	24	20	15	0	10	9.54	0.11
7	25	10	15	0	20	10.21	0.29
15	26	10	0	20	10	10.92	1.02
6	27	10	15	20	0	8.40	0.55
20	28	20	15	20	10	11.76	0.27
24	29	10	30	10	20	10.64	0.38

The highest responses of OD_600_ and conversion efficiency were highlighted in italics

Here, Y represented OD_600_. The statistical significance of Eq. (1) was estimated by *F*-value and the ANOVA for response surface quadratic model (Additional file 1: Table S2). The *F*-value of model was 6.55 which implied the model for OD_600_ was significant. The low probability *P* value of model was 0.0006 which mean there was only 0.06% chance that the *F*-value of model may occur large owing to noise. The “Lack of Fit F-value” of 0.45 implied the Lack of Fit is not significant relative to the pure error. There was 85.90% chance that a “Lack of Fit F-value” this large could occur due to noise. The R^2^ value of 0.8676 gives 86.76% variability in the response of OD_600_, and about 13.24% total variation cannot be explained by the model. The value of adequate precision was 9.489, a ratio greater than 4, which showed that the polynomial quadratic model was of an adequate signal, and could be used to navigate the design space. According to the principle that the smaller the *P*-value, the more significant the corresponding coefficient, it could be seen from Additional file 1: Table S2 that two linear coefficient (factors A and D) and two quadratic coefficients (A^2 and D^2) were highly significant, indicating that glucose and yeast extract played important roles in the cell growth.

2 $$\begin{aligned} {\text{Y}} = & 4.912 - 7.07 * {\text{A}} + 0.81 * {\text{B}} - 0.32 * {\text{C}} + 1.04 * {\text{D}} - 2.61 * {\text{A}}*{\text{B}} \\ & + 1.71 * {\text{A}}*{\text{C}} - 2.50 * {\text{A}}*{\text{D}} - 0.075 * {\text{B}}* {\text{C}} - 0.53 * {\text{B}}*{\text{D}} \\ & + 0.95 * {\text{C}}*{\text{D}} + 6.81 * {\text{A}}*{\text{A}} - 1.34 * {\text{B}}*{\text{B}} + 1.16 * {\text{C}}*{\text{C}} + 1.48 * {\text{D}}*{\text{D}} \\ \end{aligned}$$

In Eq. (2), Y was the conversion efficiency. The statistical significance of Eq. (10) was estimated by *F*-value and ANOVA as given in Additional file 1: Table S3. The *F*-value of the model for conversion efficiency was 8.72, indicating this model was significant. The probability *P*-value of the model and the Lack of Fit was 0.0001. The R^2^ value of 0.8971 gives 89.71% variability in the response of conversion efficiency, and about 10.29% total variation cannot be explained by the model. The value of adequate precision was 10.624. The *P*-value of factor A in the model was below 0.0001, suggesting that factor A (glucose) was extremely significant correlated with the conversion efficiency but the other three factors were not significant (*P*-value > 0.05) (Additional file 1: Table S3).

### Results of Box–Behnken Design for medium composition optimization

**Fig. 2:**
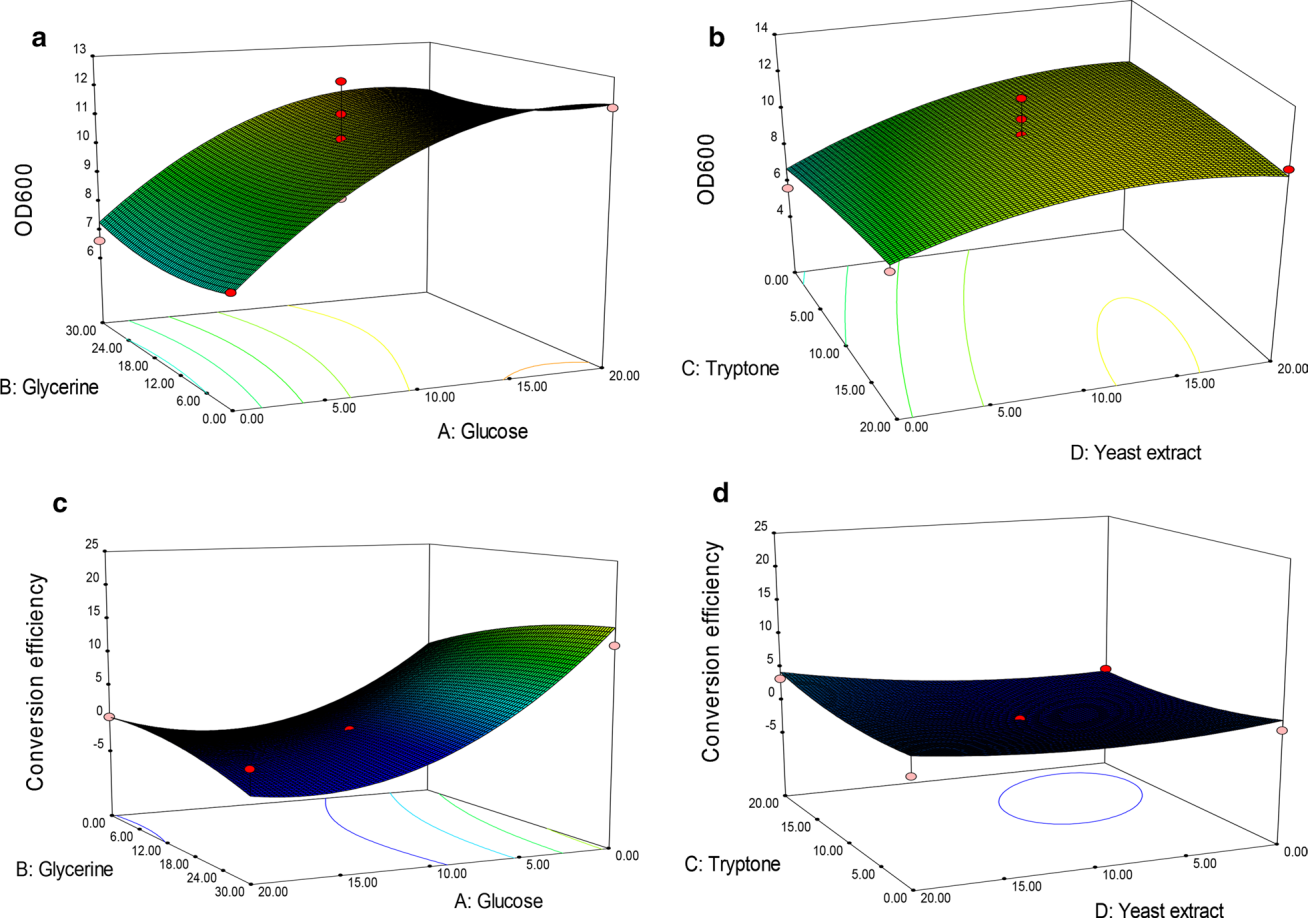
Results of response surface methodology for OD_600_ and TUDCA conversion efficiency. **a**, **b** Results of RSM for OD_600_. **c**, **d** Results of RSM for conversion efficiency

The 3D graphs of response surface representation intuitively showed the relation between the response and experimental levels of each variable, which was helpful to analyze the kind of interaction among variables to deduce the optimum medium composition. As shown in Fig. 2a, b, and Additional file 1: Table S2, factors A and D had a greater impact on OD_600_ than B and C did. Both were positively correlated with OD_600_; whereas factor A had a negative significant effect on conversion efficiency (Additional file 1: Table S3; Fig. 2c, d). Meanwhile, the maximal OD_600_ and conversion efficiency was predicted for the optimized medium by setting three stars for conversion efficiency and one star for OD_600_ due to the conversion of TUDCA was the primary goal of the study. A total of 55 solutions indicating the suggested medium composition were given and the top ten was listed in Table 2. The selected one composed of 28.43 mL/L of glycerol and 20 g/L of yeast extract without glucose and tryptone (Table 2). This solution was chosen together with the original ingredients of M9 minimal medium (6.78 g/L of Na_2_HPO_4_, 3 g/L of KH_2_PO_4_, 0.5 of g/L of NaCl, 1 g/L of NH_4_Cl, 2 mM of MgSO_4_, 0.1 mM of CaCl_2_) as the optimized medium for further study to verify the results of BBD and determine the accuracy of the model.

**Table 2:** Solutions given by Box–Behnken Design for medium composition

Solution no.	Suggested concentration (g/L)				Expected		Desirability
	Glucose	Glycerol	Tryptone	Yeast extract	OD_600_	Conversion efficiency	
*1*	*0.00*	*28.43*	*0.00*	*20.00*	*7.45*	*22.7%*	*0.23*
2	0.01	27.87	0.00	20.00	7.44	22.7%	0.23
3	0.00	26.99	0.00	20.0	7.41	22.65	0.23
4	0.00	30.00	0.00	19.37	7.55	22.45	0.22
5	0.00	24.17	0.00	19.98	7.36	22.44	0.22
6	0.17	29.84	0.01	20.0	7.57	22.30	0.22
7	0.20	30.00	0.00	20.0	7.58	22.23	0.22
8	0.00	30.00	0.00	18.70	7.60	22.13	0.22
9	0.00	21.04	0.04	19.97	7.34	22.09	0.22
10	0.00	30.00	2.40	20.00	7.42	22.00	0.22

The top 10 of 55 solutions were listed and the selected one was highlighted in italics

On experimentation using the optimized medium, the observed response of OD_600_ and TUDCA conversion efficiency were 7.21 and 19.74%, respectively, very close to the corresponding predicted values, 7.45 and 22.72%, respectively (Table 2) and much higher than the control (PBS-M9 medium), 2.15 and 1.70%, respectively. These results proved that the developed model was accurate and the optimized medium was efficient and workable for improvement of both cell density and TUDCA formation.

### Results of IPTG induction optimization for TUDCA formation

**Fig. 3:**
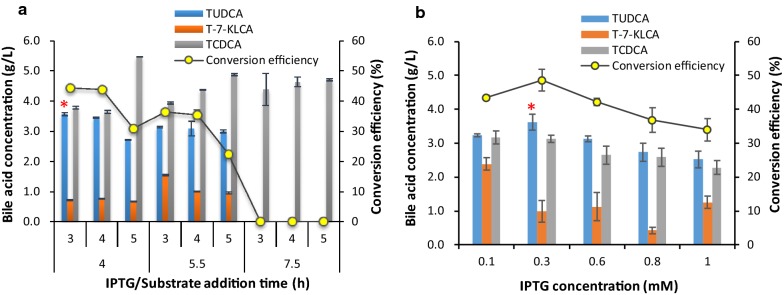
Results of IPTG induction optimization for TUDCA conversion. **a** Results of IPTG and substrate addition time. **b** Results of IPTG concentration. A red asterisk above the column indicated the optimal conditions

IPTG induction was optimized using the optimized medium mentioned above in shake flasks. The addition time points of IPTG and substrates were firstly explored by adding 1.0 mmol/L of IPTG. By addition of IPTG after sub-culturing the bacteria for 4 h (logarithmic prophase) and supplementing substrates 3 h later, the highest TUDCA titer and conversion efficiency, respective 3.57 ± 0.05 g/L and 44.20 ± 0.01% (Fig. 3a), was obtained. The concentration of TUDCA was similar to this result when supplying substrates 4 h later but was less when delaying substrate addition time to 5 h (Fig. 3a). The later IPTG addition time 5.5 h and 7.5 h, corresponding to logarithmic metaphase and logarithmic late stage, led to less TUDCA than earlier IPTG addition, no matter which substrate addition time was combined, correspondingly. These results indicated that it was better for TUDCA conversion to add IPTG at the logarithmic prophase (4 h) and load substrate 3–4 h later when using the optimized medium.

IPTG concentration was then explored using the optimal addition time. When IPTG increased from 0.1 mmol/L to 1.0 mmol/L, the titer of TUDCA and conversion efficiency increased firstly then reduced (Fig. 3b). The highest TUDCA titer was 3.61 ± 0.23 g/L and the highest conversion efficiency was 48.63 ± 3.16% when 0.3 mmol/L of IPTG was supplemented, indicating that 0.3 mmol/L of IPTG was better for TUDCA formation.

### Effect of feedstock composition and feeding process on the biotransformation

**Fig. 4:**
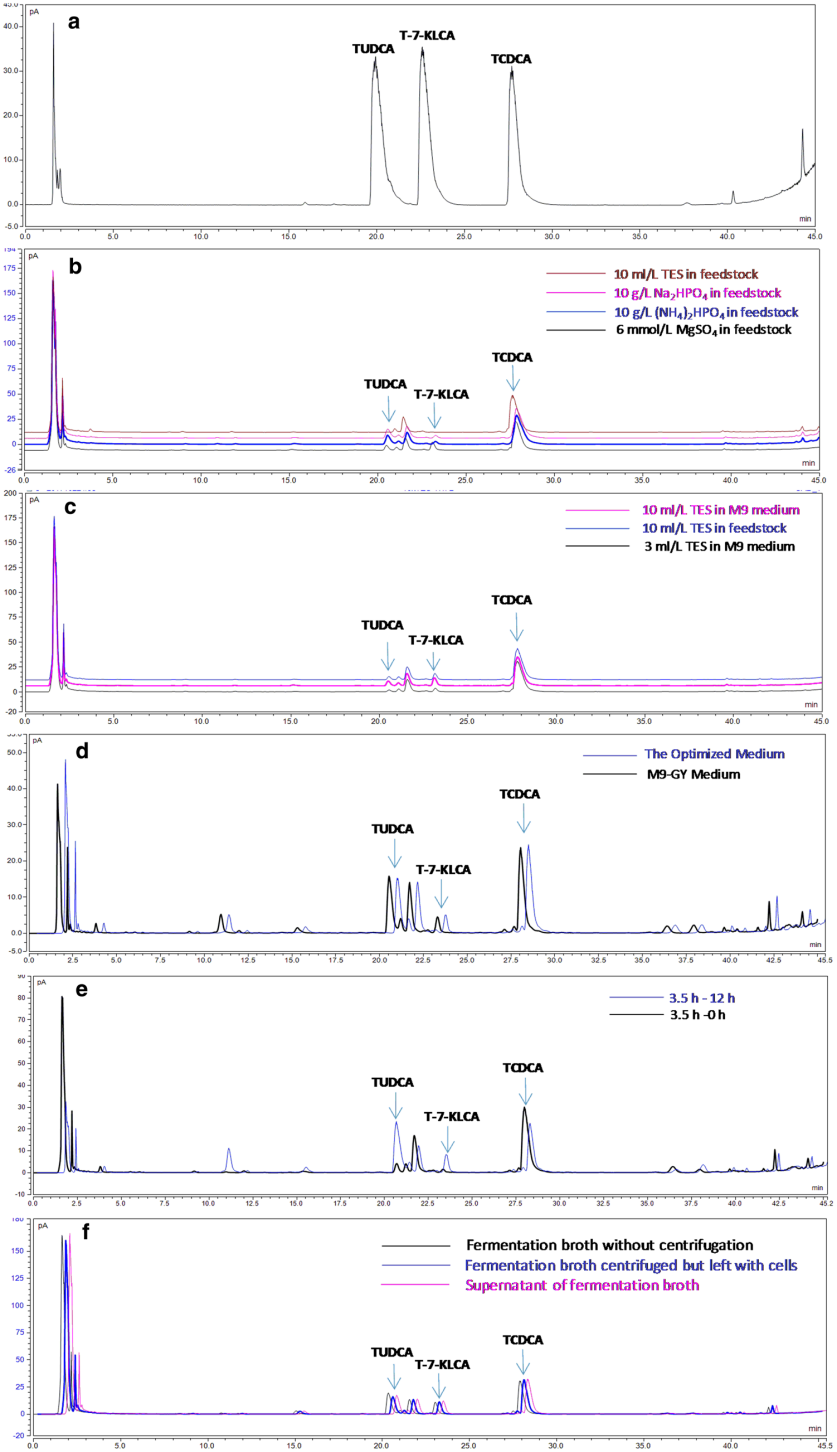
Typical HPLC profiles of bile acids from fermentation optimization experiments. **a** Reference standards of TUDCA, T-7-KLCA, and TCDCA. **b**, **c** Results of feedstock optimization. **d** Results of M9-GY medium compared with the optimized medium. **e** Results of 12 h static process following 3.5 h stirring fermentation compared with 3.5 h stirring fermentation alone. **f** Results of separating cells from fermentation broth on TUDCA conversion during the static process

Feedstock containing 20–200 mL/L of glycerol and 40–60 g/L of yeast extract was firstly fed during the fermentation process. As a result, the OD_600_ value of *E. coli* BL-pα_1_β_2_ strain was up to 13 ± 2 at platform stage. When feedstock containing glycerol and yeast extract supplemented with 10 mL/L of trace element solution (TES) was used, the OD_600_ sharply increased to 120 ± 10. Yet, the TUDCA conversion was less than those using Na_2_HPO_4_ or (NH_4_)_2_HPO_4_ as displayed in their typical HPLC profiles (Fig. 4b). Then, the trace elements were supplemented in the initial media to do fermentation without feeding step and similar result was got (Fig. 4c). That was high bacterial density but low TUDCA concentration. These results demonstrated that trace elements were beneficial to cell growth while Na_2_HPO_4_ and (NH_4_)_2_HPO_4_ were conducive to TUDCA formation. It also indicated that feeding step might be unnecessary in this study. Therefore, trace elements and the feeding step were omitted in subsequent experiments. Instead, 3 g/L of (NH_4_)_2_HPO_4_ was added in the optimized medium forming the M9-GY medium for the next batch fermentation, considering both the effect of Na_2_HPO_4_ and (NH_4_)_2_HPO_4_ on TUDCA formation and the original salt ingredients of M9 minimal medium. The OD_600_ and TUDCA conversion efficiency using M9-GY medium were 11.32 and 24.79%. Both were higher than that using the optimized medium, 7.21 and 19.74%, respectively. This result indicated that M9-GY medium was more favorable for both cell growth and TUDCA conversion efficiency than the optimized medium. Meanwhile, by comparing Fig. 4c, d, it could be seen that more TUDCA was produced using the optimized medium without TES and feeding step, which demonstrated that TES and feeding step was unfavorable for the yield of TUDCA in this study.

### Results of deep-tank static process optimization

**Fig. 5:**
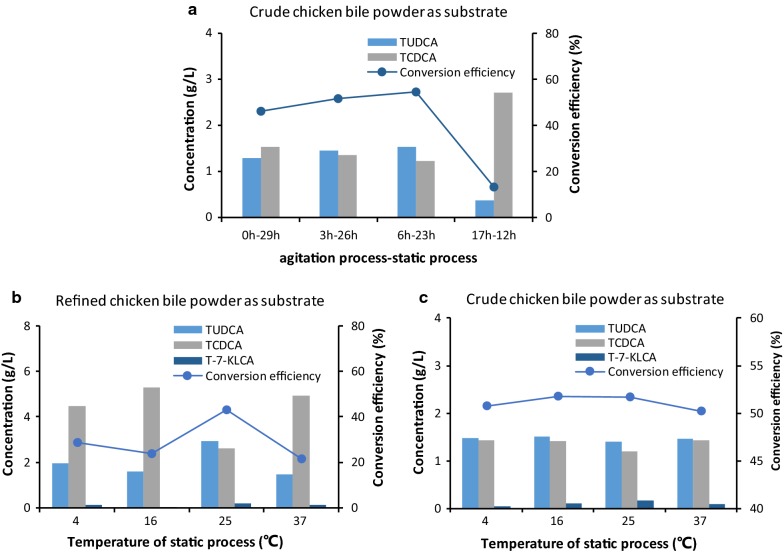
Results of deep-tank static process optimization. **a** Duration optimization of agitation and static processes. **b** Temperature optimization using refined chicken bile powder as substrate. **c** Temperature optimization using crude chicken bile powder as substrate

Using the M9-GY medium, we firstly did the fermentation in fermenters referred to the optimal fermentation time obtained in shake flasks where the optimal conditions was keeping the fermentation at 25 °C for 3–12 h in PBS-M9 media [22] but didn’t get good results (Table 3, No. 2–4). The occasional finding that keeping fermentation broth still for 12 h after 3.5 h stirring fermentation promoted TUDCA conversion (Fig. 4e) inspired us to explore a deep-tank static process following the batch fermentation program. As shown in Fig. 5a, among the four groups, 6 h stirring fermentation (the agitation process) then keeping static (the static process) for 23 h led to the highest TUDCA titer and conversion efficiency, 1.54 g/L and 54.54%, respectively. 3 h-agitation and 26 h-static (3 h–26 h combination) yielded 1.06 g/L of TUDCA with 51.68% conversion efficiency. The 17 h-12 h combination had the lowest TUDCA content with the lowest conversion efficiency, 0.14 g/L and 13.37%, respectively. These results suggested that the duration of both agitation and static processes had significant influence on the TUDCA biotransformation.

**Table 3:** Results of agitation fermentation coupled with static process

No.	Time agitation process-static process	Tube volume (mL)	TUDCA (g/L)	T-7-KLCA (g/L)	TCDCA (g/L)	Ratio of TUDCA to TCDCA	TUDCA conversion efficiency (%)
1	0 h–0 h	▲	0.00	0.00	1.10	0.00	0.00
2	3.5 h–0 h	▲	0.04	0.00	1.06	0.04	3.55
3	6.5 h–0 h	▲	0.07	0.01	1.00	0.07	6.05
4	16.5 h–0 h	▲	0.18	0.04	0.89	0.20	15.94
5	0 h–16.5 h	2	0.15	0.02	0.93	0.16	13.23
6		15	0.27	0.04	0.79	0.34	24.24
7		50	0.26	0.01	0.83	0.31	23.30
8	0 h–22 h	2	0.19	0.05	0.86	0.22	17.22
9		15	0.30	0.03	0.78	0.40	27.73
10		50	0.28	0.06	0.76	0.36	25.21
11	0 h–48 h	2	0.19	0.05	0.86	0.22	17.01
12		15	0.30	0.06	0.74	0.41	27.22
13		50	0.21	0.16	0.73	0.28	18.92
14	3.5 h–13 h	2	0.54	0.03	0.52	1.05	*49.58*
15		15	0.56	0.04	0.50	1.11	*50.76*
16		50	0.58	0.04	0.48	1.20	*52.70*
17	3.5 h–18.5 h	2	0.54	0.06	0.49	1.10	*49.49*
18		15	0.53	0.07	0.50	1.05	*47.94*
19		50	0.52	0.07	0.51	1.02	*47.10*
20	3.5 h–44.5 h	2	0.50	0.09	0.51	0.98	*45.52*
21		15	0.51	0.09	0.50	1.02	*46.42*
22		50	0.51	0.10	0.49	1.05	*46.60*
23	6.5 h–10 h	2	0.52	0.07	0.51	1.02	*47.41*
24		15	0.51	0.06	0.52	0.98	*46.74*
25		50	0.52	0.06	0.52	1.00	*47.20*
26	6.5 h–15.5 h	2	0.42	0.07	0.61	0.69	38.25
27		15	0.41	0.08	0.61	0.67	37.36
28		50	0.43	0.06	0.60	0.71	39.25
29	6.5 h–41.5 h	2	0.50	0.09	0.51	0.96	*45.19*
30		15	0.49	0.09	0.52	0.94	44.38
31		50	0.51	0.09	0.50	1.03	*46.66*
32	16.5 h–5.5 h	2	0.19	0.06	0.85	0.22	17.25
33		15	0.20	0.07	0.83	0.24	17.83
34		50	0.24	0.09	0.76	0.32	22.28
35	16.5 h–31.5 h	2	0.22	0.09	0.80	0.27	19.62
36		15	0.32	0.09	0.69	0.47	29.21
37		50	0.32	0.09	0.69	0.46	28.94

**▲**These samples were directly taken from fermenters without static process. TUDCA conversion efficiency above 45.00% was highlighted in italics

Further studies with 15 different “agitation and static processes” combinations revealed the ideal ratio of TUDCA to TCDCA did not appear by mixing fermentation alone (Table 3, No. 2–4) or by static process alone (Table 3, No. 5–13). The best results, TUDCA conversion efficiency above 45%, were obtained from those combinations of 3.5 h and 6.5 h of stirring fermentation— with varied duration of static process (Table 3, No. 14–25, 29, 31). These results indicated that agitation process ranging from 3.5 h to 6.5 h and static process extending from 10 to 44.5 h was benefit for TUDCA formation.

Considering the economic costs, we conducted temperature optimization for static process by setting 3.5 h for agitation fermentation coupled with 12 h static process (3.5 h-12 h combination) when refined chicken bile powder was used as substrate and 5 h agitation fermentation with 12 h static process (5 h-12 h combination) when crude chicken bile powder was used as substrate. As shown, when using refined chicken bile powder, keeping static process at 25 °C resulted in the highest TUDCA titer and conversion efficiency, 1.12 g/L and 43.13%, respectively. The ratio of TUDCA to TCDCA was 1.12. Keeping at 4 °C, 16 °C, and 37 °C led to less TUDCA and lower conversion efficiency, below 1.95 g/L and 28.62%, respectively (Fig. 5b). The ratios of TUDCA to TCDCA in these groups were less than 0.44. This result suggested 25 °C was the optimal temperature for static process using refined chicken bile powder as substrate. When using crude chicken bile powder and holding static process at 4 °C, 16 °C, 25 °C, and 37 °C, the TUDCA concentration was respective 1.47 g/L, 1.50 g/L, 1.39 g/L, and 1.46 g/L and TUDCA conversion efficiency was 50.77%, 51.80%, 51.65%, and 50.20%, respectively (Fig. 5c). The ratios of TUDCA to TCDCA ranged from 1.01 to 1.17, all above 1.0. This result indicated that the temperature of static process had little effect on TUDCA formation for crude chicken bile powder substrate and 25 °C might be the best choice in view of convenience.

Taken together, it suggested that coupling with 12 h-static process at 25 °C would help convert TCDCA to ideal ratio of TUDCA through fermentation of the engineered *E. coli* BL-pα_1_β_2_ strain, either using refined chicken bile powder or using crude chicken bile powder as substrates.

### Effect of dissolved oxygen on TUDCA production in static process

The result that coupling with a deep-tank static process significantly promoted the biotransformation from TCDCA to TUDCA interested us in exploring the possible explanation. The observation that the indicator value of the dissolved oxygen electrode increased from 30 to more than 70 after keeping still overnight gave a hint that it might be connected to the dissolved oxygen. To verify this speculation, 2 mL of fermentation broth was separately transferred into 2 mL, 15 mL, and 50 mL of tubes and kept at 25 °C overnight in 11 different “agitation and static processes” combinations to analyze the TUDCA production. As shown in Table 3, four combinations, 3.5 h–13 h (No. 14–16), 3.5 h–18.5 h (No. 17–19), 3.5 h–44.5 h (No. 20–22), and 6.5 h–10 h (No. 23–25), had relatively high TUDCA conversion efficiency. Among these combinations, TUDCA conversion varied with the volume of tubes but the difference was not significant. For example, the TUDCA conversion efficiency of 3.5 h–13 h group was 49.58%, 50.76%, and 52.70% when keeping the broth static in 2 mL, 15 mL, and 50 mL tubes, respectively (Table 3, No. 14–16). Meanwhile, the TUDCA conversion efficiency varying with tube volumes in different combinations was irregular. For instance, in 3.5 h–13 h group, the TUDCA conversion increased from 49.58 to 52.70% with the tube volume changed from 2 to 50 mL, whereas in 3.5 h–18.5 h group, the TUDCA conversion decreased from 49.49 to 47.10% with the tube volume changed from 2 to 50 mL. These results indicated that dissolved oxygen might influence the biotransformation via a complicated mechanism.

### Results of separating cells from fermentation broth for TUDCA conversion

As shown in Fig. 4f, TUDCA in fermentation broth without centrifugation was slightly higher than that in supernatant and that in fermentation broth centrifuged but left with cell pellets, while TCDCA in the control sample was a little lower than that in the other two samples. This result indicated that separating cells from the broth had a little effect on TUDCA conversion. Nevertheless, compared with the sample without static process (Table 3, No. 2–4), TUDCA in the three samples including the supernatant separated from cells was all increased obviously, demonstrating that there were active 7α-HSDH and 7β-HSDH enzymes released from the cells into the broth after a period stirring fermentation and these enzymes catalyzed the reaction from TCDCA to TUDCA during the static process. It also implied that separation of cells from fermentation broth was not necessary to promote TUDCA conversion.

### Preparation and chemical analysis of products via the optimized fermentation process

**Fig. 6:**
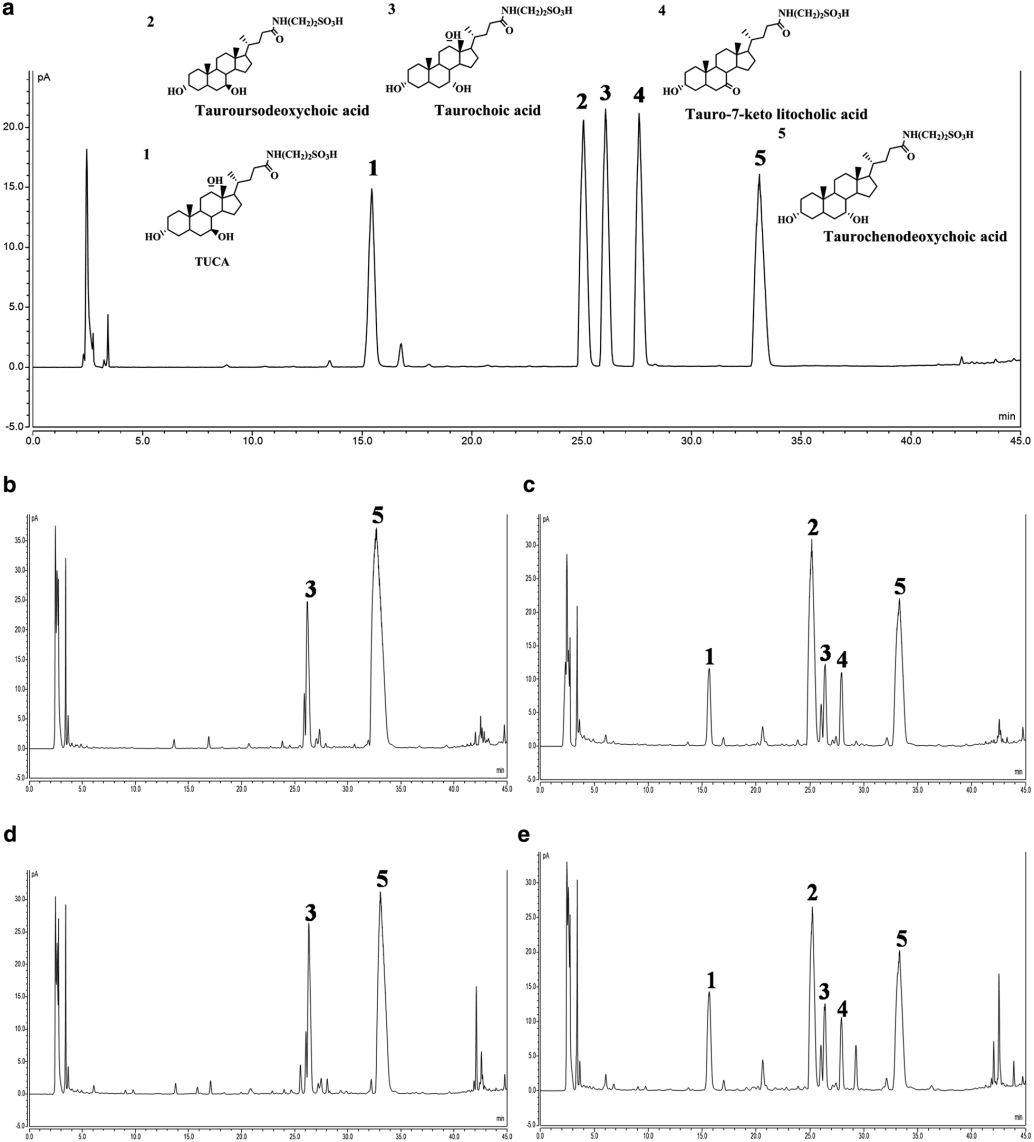
Typical HPLC profiles of bile acids in prepared products and corresponding substrates. **a** Reference standards. **b** Refined chicken bile powder (RCBP). **c** Products from RCBP. **d** Crude chicken bile powder (CCBP). **e** Products from CCBP. Compound 1: tauroursocholic acid (TUCA). Compound 2: tauroursodeoxycholic acid (TUDCA). Compound 3: taurocholic acid (TCA). Compound 4: tauro-7-keto lithocholic acid (T-7-KLCA). Compound 5: taurochenodeoxycholic acid (TCDCA)

Utilizing M9-GY media and the optimized stirring fermentation coupled with the deep-tank static process, three batches of refined chicken bile powder (30 g) and crude chicken bile powder (35 g) were separately added as substrates to do biotransformation and preparation, yielding 29.35 ± 2.83 g and 30.78 ± 3.04 g of dry powder products (Table 4; Additional file 1: Fig. S4), respectively. When using refined chicken bile powder as substrate, the content of TUDCA was 35.85 ± 3.85% compared with 24.10 ± 2.65% of TCDCA (Table 4), a ratio of 1.49 ± 0.14, in the prepared powder products. Five principal bile acids took 74.85 ± 6.94% in the products in contrast to 72.80% in substrates (Table 4). When using crude chicken bile powder as substrate, the percentage of TUDCA was 27.14 ± 4.23% and that of TCDCA was 17.48 ± 1.68% (Table 4), a ratio of 1.55 ± 0.19. The proportion of five principal bile acids was 66.79 ± 6.96% compared with 58.30% in crude chicken bile powder (Table 4). Both the percentage of TUDCA and TCDCA and the ratio of these two bile acids were similar to that in drainage bear bile powder in market. The typical HPLC profiles of substrates and corresponding products were exhibited in Fig. 6. Considering the more complicated preparation process of refined chicken bile powder than that of crude chicken bile powder, it should be better using crude chicken bile powder as substrate for the preparation of TUDCA biotransformation products.

**Table 4:** Results of chemical analysis of products prepared from fermentation broth using optimal agitation and static processes

Substrates/products	TUDCA (%)	TCDCA (%)	T-7-KLCA (%)	TUCA (%)	TCA (%)	UDCA (%)	TUDCA + TCDCA (%)	Five bile acids (%)	Moisture (%)	Endotoxin (EU/mL)	Weight (g)
Refine chicken bile powder (RCBP)^a^	0.00	65.00	0.00	0.00	7.40	0.40	65.00	72.80	2.8 ± 0.2	0.001	30.00
Products from RCBP^b^	35.85 ± 3.85	24.10 ± 2.65	3.54 ± 0.48	8.69 ± 2.75	2.45 ± 0.49	0.22 ± 0.08	58.91 ± 5.77	74.85 ± 6.94	4.68 ± 1.11	0.003 ± 0.001	29.35 ± 2.83
Crude chicken bile powder (CCBP)^a^	0.00	45.00	0.00	0.00	13.00	0.30	45.00	58.30	4.1 ± 0.4	0.0010	35.00
Products from CCBP^b^	27.14 ± 4.23	17.48 ± 1.68	4.29 ± 0.54	13.06 ± 1.20	2.37 ± 0.15	2.44 ± 0.30	43.91 ± 5.33	66.79 ± 6.96	2.26 ± 0.28	0.003 ± 0.000	30.78 ± 3.04

^a^Data of refine chicken bile powder and crude chicken bile powder were separately from one batch

^b^Data of products from RCBP and CCBP were from three batches, respectively

**Fig. 7:**
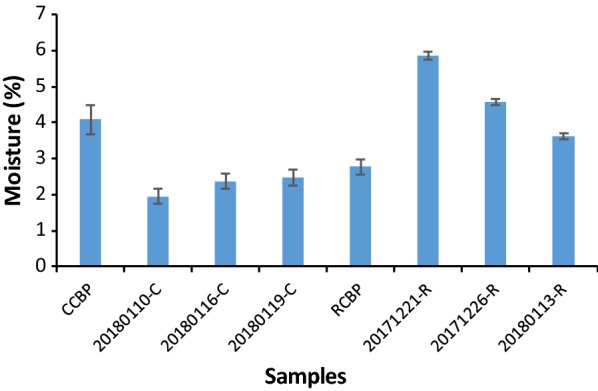
Moisture in chicken bile powder and corresponding products

The moisture and endotoxin of the prepared products were measured. As shown, moisture content of the prepared products from refined chicken bile powder was 4.68 ± 1.11 and that from crude chicken bile powder was 2.26 ± 0.28 (Table 4; Fig. 7). The endotoxin of all the samples was lower than 0.015 EU/mL (Table 4) calculated according to the standard curve of endotoxin (Additional file 1: Fig. S3).

**Fig. 8:**
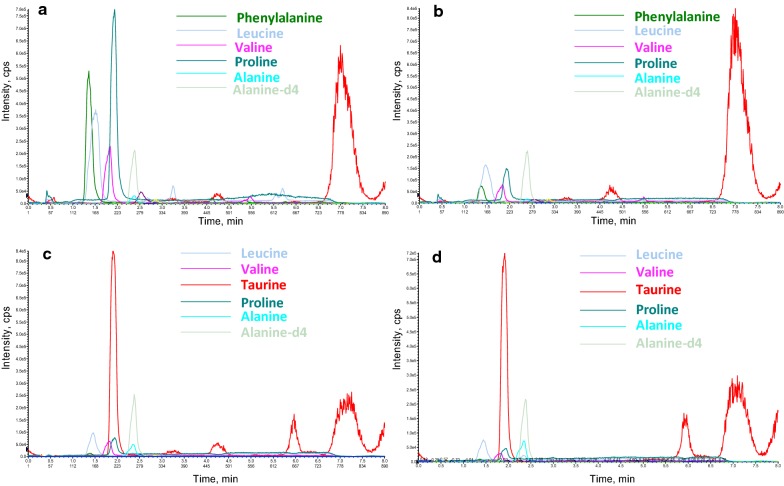
Typical mass chromatogram of amino acids in prepared products from chicken bile powder. **a** Amino acids in products from refined chicken bile powder. **b** Amino acids in products from crude chicken bile powder. **c** Amino acids in products from RCBP when static process at 37 °C. **d** Amino acids in natural bear bile powder sample

Amino acids in the prepared products were also monitored. Except for taurine, the other five amino acids from the prepared products, leucine, valine, proline, alanine, and alanine-d4, were present at a much higher level than that from drainage bear bile powder (Fig. 8). Besides, all products had a considerable amount of phenylalanine. The level of these amino acids in products produced from refined chicken bile powder was higher than the corresponding one produced from crude chicken bile powder, possibly due to amino acids were enriched in refined chicken bile powder. Interestingly, when the coupled static process was set at 37 °C, there was nearly equal amount of taurine in the products from refined chicken bile powder to that in drainage bear bile powder (Fig. 8). This result indicated that keeping the static process at 37 °C was not conductive to the production of TUDCA but benefit for the formation of taurine which was the main amino acid component in drainage bear bile powder.

## Discussion

An efficient Large-scale fermentation process is important for industrial application of synthetic biology approach to produce value-added natural products. In previous work, we recreated a two-enzyme catalyzing biosynthetic pathway of TUDCA in *E. coli* which has the potential to produce bear bile powder substitute from cheap and readily available chicken bile powder contributing to the bidirectional oxidative and reductive reactions of 7α-HSDH and 7β-HSDH [22]. In that work, we used a phosphate buffer solution modified M9 minimal medium (PBS-M9) and a two-step fermentation procedure to concentrate bacteria to high density and promote the TUDCA production in shake flasks [22]. Replacing two-step fermentation with one-step method is essential to decrease the cost for chemical production in industrial scale [27]. The challenges of one-step fermentation is to improve the cell growth and the production of target compounds as well [27, 28]. In order to increase the lactic acid production in one-step fed-batch fermentation, Fu et al. used as high as 100 g/L of glucose in the initial media and fed glucose as feedstock during the fermentation process as well [28]. Since first fermentation using PBS-M9 medium in the fermenter got low cell density (OD_600_ equal to 2.15) and low TUDCA conversion efficiency (1.70%), we then tried several rich media including LB, 2-YT, and SOC but failed to get appropriate TUDCA conversion (Data not shown).

Taking the advantage of interactive analysis of RSM, we then tried to optimize the carbon and nitrogen sources favorable for bacterial growth including glucose, glycerol, tryptone, and yeast extract [29, 30] based on M9 medium using cell density and product conversion efficiency as two responses. RSM is one of the most popular methods applied in biotechnology for fermentation optimizations [31–33]. It provides an alternative way to analyze systems where the mathematical relationship between the parameters and the responses are unknown and to reflect the complex nonlinear relationships between independent variables and responses of the system [34]. By using this method, an optimal medium composed of glycerol and yeast extract was predicted (Table 2) and experimentally verified with improved TUDCA production (Fig. 4d), which indicated that optimizing medium components with RSM was flexible to balance the cell growth and the metabolic flux for the recreated multi-gene pathway in host cells.

Phosphate is involved in many biochemical reactions and plays an important role in microbial growth and product synthesis [35–37]. Phosphate scarcity is often applied to limit cell growth and improve heterologous protein expression in microbial production processes [36, 38]. It means low phosphate would result in low cell density but high catalytic capability of individual cell if heterologous enzymes were aimed to express in the microbial cells. Yet, high cell density cultivation is usually required for high yield of target compounds from fermentation of microorganisms engineered with reconstructed heterologous biosynthetic pathway [39, 40]. In this study, feeding 10 g/L of Na_2_HPO_4_ or (NH_4_)_2_HPO_4_ improved TUDCA conversion (Fig. 4b) and supplementation of 3 g/L of (NH_4_)_2_HPO_4_ in the medium increased both TUDCA conversion (Fig. 4d) and cell density, indicating that high production can be obtained by adjusting the phosphate concentration to equilibrate the cell growth and the catalytic ability of individual cell during the fermentation process.

The result that a period static process following 3.5 h–6.5 h stirring fermentation promoted TUDCA conversion was an interesting finding in present study (Table 3, Fig. 4f). Most likely, the heterologous expressed 7α-HSDH and 7β-HSDH released into the media contributed to the improvement of TUDCA conversion from TCDCA during static process, as indicated in Results (Fig. 4f). Both 7α-HSDH and 7β-HSDH used in the construction were not secretory proteins [22–24]. Their release into the fermentation broth was possibly due to bacterial autolysis caused by the unfavorable growth conditions [41] during the static process, as indicated by the increase of the dissolved oxygen value from 30 to about 70. Moreover, the conversion efficiency was still above 45% after keeping the broth static for more than 40 h following mixing fermentation (Table 3, No. 14–34). It could be inferred that the 7α-HSDH and 7β-HSDH released into the broth kept its oxidative or reductive activity as long as 44.5 h. This observation was different from our work in shake flasks where the dominant catalytic activity of “β2” was first reductive then turned to oxidation at around 12 h [22]. As for the effect of dissolved oxygen on TUDCA conversion during static process, it was probably that dissolved oxygen affected the enzyme activities since both 7α-HSDH and 7β-HSDH were oxygen-dependent enzymes [22–24].

It should be mentioned that the chicken bile powder used in this study is abundant in TCDCA but not in pure TCDCA. There are other compositions like TCA which was also transformed to tauroursocholic acid (TUCA) during the fermentation. The pharmacological evaluation of these products is in progress.

## Conclusion

In summary, through optimizing the fermentation medium by RSM and developing a special deep-tank static process, we achieved 29.35 ± 2.83 g and 30.78 ± 3.04 g of dry powder products respectively from 30 g of refined chicken bile powder and 35 g of crude chicken bile powder. These products contained 35.85 ± 3.85% and 27.14 ± 4.23% of TUDCA at a ratio of 1.49 ± 0.14 and 1.55 ± 0.19 to TCDCA, a similar proportion to the medicinal material drainage bear bile powder. This study provided a practical and environment friendly industrialized process for producing artificial substitute of bear bile powder from cheap and readily available chicken bile powder using microbial cell factory (Additional file 1: Fig. S5). It also put forward a new strategy to advance enzyme-catalyzing reactions in fermentation by coupling with deep-tank static process.

## Materials and methods

### Bacterial strains and chemical regents

The engineered *E. coli* BL21star™ (DE3) strain BL-pα_1_β_2_ used in this study was created in our lab [22] and kept under − 80 °C freezer. Crude chicken bile powder (containing 45.11 ± 0.3% of TCDCA) and refined chicken bile powder (containing 64.78 ± 0.3% of TCDCA) were kindly provided by Shanghai Kaibao Pharmaceutica Co. Ltd., China. Resin D101 was purchased from Wuhan Weiqiboxin Biotechnology Co. Ltd., China. All the other chemicals in this study were from Sinopharm Chemical Reagent Co. Ltd., China.

### Medium optimization by response surface methodology (RSM)

Box–Behnken Design (BBD) of RSM was applied to do medium optimization experiment based on the inorganic salt composition of M9 minimal medium in shake flasks with the aim to improve the cell density and TUDCA conversion efficiency. Four independent factors, glucose, glycerol, tryptone, and yeast extract, respectively designed as factor A, B, C, and D, were investigated at three different levels. Factors A, C, and D were divided into 0, 10, 20 g/L grades and B was divided into 0, 15, 30 mL/L grades with five repetitions at the central point using cell density (OD_600_) of the finally broth and TUDCA conversion efficiency as two responses (Table 1). The media used for optimization experiment were named as designed media.

The conversion efficiency in this study was indicated as the TUDCA yield calculating according to the Eq. (3) as follows:

3 $${\text{The conversion efficiency}} = \frac{{{\text{Total amount of TUDCA generated }}\left( {\text{g}} \right)}}{{{\text{Total amount of TCDCA added }}\left( {\text{g}} \right)}} \times 100\% .$$

To get the response data from the BBD, fermentation of *E. coli* BL-pα_1_β_2_ was carried out in shake flasks applying the procedure described above. The experimental response data were then supplied to Design-Expert 8.0.6.1 software and fitted with a second-order polynomial Eq. (4) as follows:

4 $$\begin{aligned} {\text{Y}} =& {\text{b}}_{ 0} {\text{ + b}}_{ 1} {\text{A + b}}_{ 2} {\text{B + b}}_{ 3} {\text{C + b}}_{ 4} {\text{D + b}}_{ 1 1} {\text{A}}^{ 2} {\text{ + b}}_{ 2 2} {\text{B}}^{ 2} {\text{ + b}}_{ 3 3} {\text{C}}^{ 2} {\text{ + b}}_{ 4 4} {\text{D}}^{ 2} \\ & {\text{ + b}}_{ 1 2} {\text{AB + b}}_{ 1 3} {\text{AC + b}}_{ 1 4} {\text{AD + b}}_{ 2 3} {\text{BC + b}}_{ 2 4} {\text{BD + b}}_{ 3 4} {\text{CD}} \\ \end{aligned}$$

where, Y is the predicted response (OD_600_ and conversion efficiency in this study), b_0_ is the model constant, b_i_, b_ii_ and b_ij_ are the linear coefficients, the squared coefficients, and the interaction coefficients, respectively.

### Fermentation procedure in shake flasks

One-step fermentation modified from Ref. [22] of recombinant *E. coli* was performed. Briefly, a single colony of *E. coli* BL-pα_1_β_2_ was inoculated in liquid LB media supplemented with 100 mg/L of ampicillin and cultured at 37 °C, 225 rpm on a horizontal shaker overnight. The pre-cultured bacteria were sub-cultured in 40 mL of designed media (for medium optimization) or PBS-M9 medium (for control) [22] at a proportion of 1:50 in 250 mL flasks and grew at 37 °C, 225 rpm for 4–5 h until OD_600_ reached 0.6–0.8. Protein expression was induced by addition of 0.3 mmol/L of isopropylthio-β-galactoside (IPTG). Several hours later, certain amount of chicken bile powder was added as substrate and cell culture further grew at 25 °C, 225 rpm for another 6 h. All cultivation in shake flasks was performed in triplicate except where noted.

### IPTG induction optimization

IPTG induction was studied in shake flasks with one-step fermentation as described above. To do that, the growth curve of the *E. coli* BL-pα_1_β_2_ strain was established. IPTG and substrate addition time was firstly studied through orthogonal experiments. 1.0 mmol/L of IPTG was added after sub-culturing the bacteria for 4 h, 5.5 h, and 7.5 h (corresponding to logarithmic prophase, logarithmic metaphase and logarithmic late stage in the bacterium growth curve Additional file 1: Fig. S1) and substrate addition time was set the following 3 h, 4 h, and 5 h, designed as 1–9 Std as listed in Additional file 1: Table S1. Then, using the optimal IPTG addition time, IPTG concentration was optimized by separately supplementing 0.1, 0.3, 0.6, 0.8 and 1.0 mmol/L of IPTG in the media.

### Data analysis

Design-Expert 8.0.6.1 software was used for the regression analysis of the experimental data obtained and statistical parameters were examined with analysis of variance (ANOVA). The fit quality of the polynomial model equation was evaluated by the determination coefficient and the significance of the model, an optimum value of parameter, was assessed with the determination coefficient and correlation coefficient. The statistical testing of the model was by *F*-value and *P*-value.

### Bacterial cultivation in stirred-tank fermenter

A single colony of *E. coli* BL-pα_1_β_2_ was inoculated in 3 mL of liquid LB media and cultured at 37 °C, 225 rpm for 12–14 h. Then, 3 mL of bacterial culture was transferred into flask containing 150 mL of 2-YT media (5 g/L of NaCl, 16 g/L of tryptone, 10 g/L of yeast extract) supplemented with 100 mg/L of ampicillin and continued culturing at 37 °C, 225 rpm for another 12–14 h. All the cultures were inoculated into the 5 L-stirred tank fermenter (Biotech-5BG, Shanghai Baoxin Bioengineering Equipment, Shanghai, China) containing 3 L of M9-GY media. Throughout the fermentation, the agitation was at 600 rpm and the airflow was at 2 L/min. In the batch phase, temperature was set at 37 °C and the pH value was kept at 7 by automatic addition of ammonia hydroxide and phosphoric acid. After culturing the bacteria for 3.5–4.5 h and OD_600_ reaching 2.5–4.0 (corresponding to logarithmic prophase of the bacterial growth curve, Additional file 1: Fig. S2), 0.3 mmol/L of IPTG was added to start protein-induced phase. During this stage, the temperature and pH value was hold at 30 °C and 6.5, respectively. Then, after 3 h, chicken bile powder was loaded as substrates to start the product formation phase extending 3–17 h accordingly. The temperature of this period was set at 25 °C and pH value was 6.5.

### Feedstock and feeding process optimization

One liter of feedstock containing 90 mL/L of glycerol and 60 g/L of yeast extract was firstly fed at 1.5 mL/min when dissolved oxygen was rebounded which means glycerol depletion and exponential growth phase began. Then 6 mmol/L of MgSO_4_, 10 g/L of (NH_4_)_2_HPO_4_, 10 g/L of Na_2_HPO_4_ and 10 mL/L of trace element solution (TES) was separately added with glycerol and yeast extract as feedstock to explore their effect on cell growth and TUDCA formation. Trace element solution consisted in 0.5 g/L of CaCl_2_, 0.18 g/L of ZnSO_4_·7H_2_O, 0.1 g/L of MnSO_4_·H_2_O, 10.05 g/L of Na_2_-EDTA, 8.35 g/L of FeCl_3_, 0.16 g/L of CuSO_4_·5H_2_O, 0.18 g/L of CoCl_2_·6H_2_O.

### Deep-tank static process optimization

A deep-tank static process coupled with stirring fermentation was exploited to promote the TUDCA biotransformation. To do that, BL-pα_1_β_2_ was cultivated in M9-GY media in the 5L-stirred-tank bioreactor as described above. The agitation process and the static process was firstly optimized by sampling the broth 0 h, 3 h, 6 h, and 17 h later by addition of 30 g/L of refined chicken bile powder as substrates and kept at 25 °C for 29 h, 26 h, 23 h, and 12 h, respectively, consisting of four different experimental groups. Group 1 represented that keeping the fermentation broth static for 29 h immediately after substrate was supplied and mixed well (0 h–29 h). Group 2 indicated the agitation process was6 h then turn to static process for 23 h (3 h–26 h). Accordingly, group 3 and group 4 were 6 h agitation fermentation coupled with 23 h static process (6 h–23 h) and 17 h agitation fermentation with 12 h static process, separately. Then, 11 combinations of agitation and static processes (as listed in the second column of Table 3) were further studied using 2 mL, 15 mL, and 50 mL tubes as the containers. The application of varied volume tubes was aimed to study the effect of dissolved oxygen on the TUDCA production as well, as described in literature, the oxygen availability in the culture could be controlled by varying the amount of headspace of cultures inside a fixed-volume containers [1, 42]. After that, the optimal combination of agitation and static processes was adopted to optimize the temperature of static process by setting at 4 °C, 16 °C, 25 °C, and 37 °C using 30 g/L of refined chicken bile powder or 35 g/L of crude chicken bile powder as substrates. Optimization experiments using fermenters were performed only once for each condition.

### Experiment of separating cells from fermentation broth for TUDCA conversion

In order to investigate how static process promoted TUDCA formation, 2 mL of fermentation broth after 6.5 h stirring fermentation was separately transferred into three centrifuge tubes. One tube of broth served as control. The other two tubes of broth were centrifuged at 5, 000 rpm to pellet the cells. Supernatant of one tube was separated from the cells by transferring into a new tube and that of the other was remained in the tube with the cell pellets. All the three tubes containing fermentation broth were allowed to stand still overnight before supplied to HPLC analyze as described above.

### Preparation of powder products from fermentation broth

Crude chicken bile powder and refined chicken bile powder were respectively used as substrates for large-scale fermentation each in triplicates. After fermentation, the broth was collected and centrifuged at 8000 rpm for 20 min to get the supernatant which was passed through pre-treated isometric resin D101 [43]. The impurities on D101 resin were firstly removed by deionized water until the eluent was colorless. Then the resin was washed with 95% ethanol to get the eluent until colorless. The eluent was concentrated through rotary evaporation at 50 °C and then filtered through 0.45 μm organic filters. The filtrate was evaporated by keeping in water bath at 50 °C to form extractum and then dried to constant weight in vacuum drying oven at 50 °C. After that, the dried solid was taken out and crushed into powder. The bile acid recovery yield in this study indicated to the main five bile acid yield calculated according to the Eq. (5) as follows:

5 $${\text{The recovery yield}} = \frac{{{\text{Total amount of five bile acids in the prepared powder }}\left( {\text{g}} \right)}}{{{\text{Total amout of five bile acids in fermentation broth }}\left( {\text{g}} \right)}} \times 100\%$$

### Bile acid analysis

The bile acids in the fermentation broth were monitored with HPLC and LC-mass spectroscopy. Before supplied to the machine, the fermentation broth was firstly centrifuged at 12,000 rpm for 1 min and the supernatant was transferred into new tube. For fermentation optimization experiments, the supernatant was directly filtered through a 0.22 μm filter and supplied to the ThermoUltiMate3000 HPLC machine. For the prepared products, 5-time the volume of supernatant of *n*-butyl alcohol was firstly added to extract bile acids followed by drying with nitrogen blowing. Then, methanol, one tenth the volume of supernatant, was added to resolve the dried bile acids and filtered through a 0.22 μm filter before supplied to HPLC or LC–MS machine.

The titer of individual bile acid was determined according to the curve of each authentic standard (Fig. 6a). The substrate concentration was expressed as the initial TCDCA concentration in the media. Conversion efficiency was expressed by the percentage of the concentration of TUDCA to the initial TCDCA concentration in the media.

6 $${\text{The content of five bile acids in the prepared powder }}\left( \% \right) = \frac{{{\text{Amount of one certain bile acid in the prepared powder}}\left( {\text{g}} \right)}}{{{\text{Amount of the prepared powder }}\left( {\text{g}} \right)}} \times 100\%$$

7 $${\text{The content of T}} - 7 - {\text{KLCA in five bile acids }}\left( \%  \right) = \frac{{{\text{Amount of T}} - 7 - {\text{KLCA in the prepared powder }}\left( {\text{g}} \right)}}{{{\text{Total amount of the five bile acids in the prepared powder }}\left( {\text{g}} \right)}} \times 100\%$$

8 $${\text{The content of TUDCA and TCDCA in five bile acids }}\left( \% \right) = \frac{{{\text{Total amount of TUDCA and TCDCA in the prepared powder }}\left( {\text{g}} \right)}}{{{\text{Total amount of five bile acids in the prepared powder }}\left( {\text{g}} \right)}} \times 100\%$$

9 $${\text{The content of total five bile acids in the perpared powder }}\left( \% \right) = \frac{{{\text{Total amount of five bile acids in the prepared powder }}\left( {\text{g}} \right)}}{{{\text{Amount of the prepared powder }}\left( {\text{g}} \right)}} \times 100\% .$$

### HPLC and LC–MS conditions

ThermoUltiMate3000 HPLC machine equipped with Waters XBridgeTM C18 column (5 μm, 4.6 mm × 150 mm) and Corona Ultra Detector (CAD) was used to analyze the bile acids in the broth from the fermentation optimization experiments. The mobile phase was acetonitrile and water (contain 0.3% formic acid with 5 mmol/L ammonium acetate) at a flow rate of 1.0 mL/min and column temperature 40 °C. HPLC gradient elution program was total 45 min with acetonitrile proportion from 20 to 90% [44]. Samples were filtered through a 0.22 μm filter and 10 μL of the filtrate was directly supplied to the HPLC machine. To quantitative analyze the bile acids from the prepared products, an Agilent Poroshell 120 EC-C18 column (2.7 μm, 4.6 mm × 150 mm) was used and the flow rate was 0.6 mL/min using n-butanol extract as the injection sample. All the other conditions were the same as described above.

Waters ACQUITYUPLCZQ2000 system (Waters Corporation, Milford, USA) was used to do LC–MS assay equipped with Waters AcquityUPLCBEH C18 column (1.7 μm, 100 mm × 1.0 mm) using methanol and 0.1% acetic acid containing 5 mmol/L of ammonium acetate as gradient elution at a flow rate of 0.6 mL/min [45]. The column temperature was 45 °C and the mass spectrometer was operated in the ESI negative mode. The capillary voltage was 2.8 kV and the cone voltage was 55 V. The source temperature was set to 120 °C, and the desolvation temperature was 350 °C, while the desolvation gas flow was set to 600 L/h, and the cone gas flow was 50 L/h [44]. The injection volume was 5 μL.

### Endotoxin and moisture assay of prepared powder products

Endotoxin was tested using Chromogenic End-point TachypleusAmebocyte Lysate (Xiamen Bioendo Technology Co. Ltd., China) according to the instructions. The moisture content was calculated according to the Eq. (10) as follows:

10 $${\text{Moisture content}} = \frac{{\left( {{\text{m}}3 - {\text{m}}2} \right)}}{{\left( {{\text{m}}5 - {\text{m}}2} \right)}} *100\%$$

The value of m2 to m5 was obtained as described below. Weighing bottles were dried in bake oven at the 105 °C for an hour and then put in dryer. Half an hour later, weighed and recorded it as m1. Repeated the above operations again and recorded the second weight as m2. If the difference between m1 and m2 is not more than 0.3 mg then we considered it as constant weight. Repeated the above operations until all weighing bottles were constant weight. When all weighing bottles were constant weight, weighed all the prepared powder samples about 300 mg and respectively recorded the weights of the weighing bottles with samples in it as m3. Then the weighing bottles with samples were treated in the same way as for the empty bottles. Half an hour later, weighed the samples as m4 and then put them in bake oven at 105 °C for 1 h and then put in dryer for half an hour. After that, weighed the samples and recorded them as m5. The difference between the m4 and m5 should no more than 0.3 mg, or else repeated the above operations.

## Additional file

**Additional file 1: Table S1.** Orthogonal experimental design of IPTG induction. **Table S2.** Analysis variance (ANOVA) results of the Box–Behnken Design for OD600. **Table S3.** ANOVA results of the Box–Behnken Design for conversion efficiency. **Fig. S1.** Growth curve of *E. coli* BL‐pα1β2 strain in shake flask. **Fig. S2.** Growth curve of *E. coli* BL‐pα1β2 strain in 5 L‐stirred tank fermenter. **Fig. S3.** Standard curve of endotoxin. **Fig. S4.** The morphology of chicken bile powder and the corresponding products. **Fig. S5.** Engineered *E. coli* factory for potential substitute of bear bile powder.
