# Supplementary material for: Large-scale production of tauroursodeoxycholic acid products through fermentation optimization of engineered Escherichia coli cell factory
Source: Microb Cell Fact. 2019 Feb 8;18:34. doi: 10.1186/s12934-019-1076-2 (PMC6368744; doi:10.1186/s12934-019-1076-2)
Supplement: Supplementary file 1 — Additional file 1: Table S1. Orthogonal experimental design of IPTG induction. Table S2. Analysis variance (ANOVA) results of the Box–Behnken Design for OD600. Table S3. ANOVA results of the Box–Behnken Design for conversion efficiency. Fig. S1. Growth curve of E. coli BL‐pα1β2 strain in shake flask. Fig. S2. Growth curve of E. coli BL‐pα1β2 strain in 5 L‐stirred tank fermenter. Fig. S3. Standard curve of endotoxin. Fig. S4. The morphology of chicken bile powder and the corresponding products. Fig. S5. Engineered E. coli factory for potential substitute of bear bile powder. [file 12934_2019_1076_MOESM1_ESM.pdf]

Additional file

Large-scale production of tauroursodeoxycholic acid products through fermentation optimization of engineered *Escherichia coli* cell factory

Yingpeng Xu, Li Yang, Shujuan Zhao*, Zhengtao Wang

The SATCM Key Laboratory for New Resources & Quality Evaluation of Chinese Medicine, The MOE Key Laboratory for Standardization of Chinese Medicines and Shanghai Key Laboratory of Compound Chinese Medicines, Institute of Chinese Materia Medica, Shanghai University of Traditional Chinese Medicine, Shanghai 201203, P.R. China

*Corresponding author. Tel: +8621-51322576, Fax: +8621-51322495, E-mail: zhaoshujuan@126.com, zhaoshujuan@shutcm.edu.cn. ORCID: 0000-0002-2269-925X

Table S1 Orthogonal experimental design of IPTG induction

Std	IPTG addition time points (h)[a]	Substrate addition time points (h)[b]
1	4	3
2	4	4
3	4	5
4	5.5	3
5	5.5	4
6	5.5	5
7	7.5	3
8	7.5	4
9	7.5	5

Note: a. Number in this line means the bacteria were sub-cultured for 4 hours, 5.5 hours, and 7.5 hours, correspondingly. b. Number in this line represents that substrates were supplemented 3 hours, 4 hours, and 5 hours later by addition of IPTG inducer.

Table S2 Analysis variance (ANOVA) results of the Box-Behnken Design for OD_{600}

Source	Sum of squares	df	Mean Square	*F*-value	*P*-value
Model	123.71	14	8.84	6.55	**0.0006**
A-Glucose	59.67	1	59.67	44.26	**< 0.0001**
B-Glycerol	0.53	1	0.53	0.39	0.5411
C-Tryptone	2.76	1	2.76	2.04	0.1748
D-Yeast extract	24.57	1	24.57	18.22	**0.0008**
AB	0.22	1	0.22	0.16	0.6918
AC	5.45	1	5.45	4.04	0.0640
AD	1.76	1	1.76	1.30	0.2730
BC	1.32	1	1.32	0.98	0.3388
BD	0.19	1	0.19	0.14	0.7104
CD	1.06	1	1.06	0.79	0.3901
A^2	15.04	1	15.04	11.15	**0.0049**
B^2	1.14	1	1.14	0.85	0.3726
C^2	0.2	1	0.2	0.15	0.7049
D^2	9.75	1	9.75	7.23	**0.0176**
Residual	18.88	14	1.35		
Lack of fit	10.02	10	1	0.45	0.859
Pure error	8.86	4	2.21		
Total	142.58	28			

R^2 = 0.8676; R_{adj}^2 = 0.7352; Adeq-Precision = 9.489

Note: The P values implied the significance of each coefficient. The smaller the value of P-value, the more significant the corresponding coefficient. P-value<0.05 means significant. The highest significant values were highlighted in bold.

Table S3 ANOVA results of the Box-Behnken Design for conversion efficiency

Source	Sum of squares	df	Mean Square	*F*-Value	*P*-value
Model	1040.08	14	74.29	8.72	**0.0001**
A-Glucose	599.44	1	599.44	70.32	**< 0.0001**
B-Glycerole	7.79	1	7.79	0.91	0.3554
C-Tryptone	1.2	1	1.2	0.14	0.7131
D-Yeast extract	13.07	1	13.07	1.53	0.2359
AB	27.31	1	27.31	3.2	0.0951
AC	11.67	1	11.67	1.37	0.2615
AD	24.94	1	24.94	2.93	0.1092
BC	0.022	1	0.022	2.62E-003	0.9599
BD	1.12	1	1.12	0.13	0.7229
CD	3.6	1	3.6	0.42	0.5264
A^2	300.54	1	300.54	35.26	**< 0.0001**
B^2	11.66	1	11.66	1.37	0.2617
C^2	8.68	1	8.68	1.02	0.3299
D^2	14.29	1	14.29	1.68	0.2163
Residual	119.34	14	8.52		
Lack of Fit	119.34	10	11.93	5.71E+005	**< 0.0001**
Pure Error	8.36E-05	4	2.09E-05		
Cor Total	1159.42	28			

R^2 = 0.8971; R_{adj}^2 = 0.7941; Adeq-Precision =10.624

Note: The *P* values implied the significance of each coefficient. The smaller the value of *P*-value, the more significant the corresponding coefficient. *P*-value<0.05 means significant. The highest significant values were highlighted in bold.

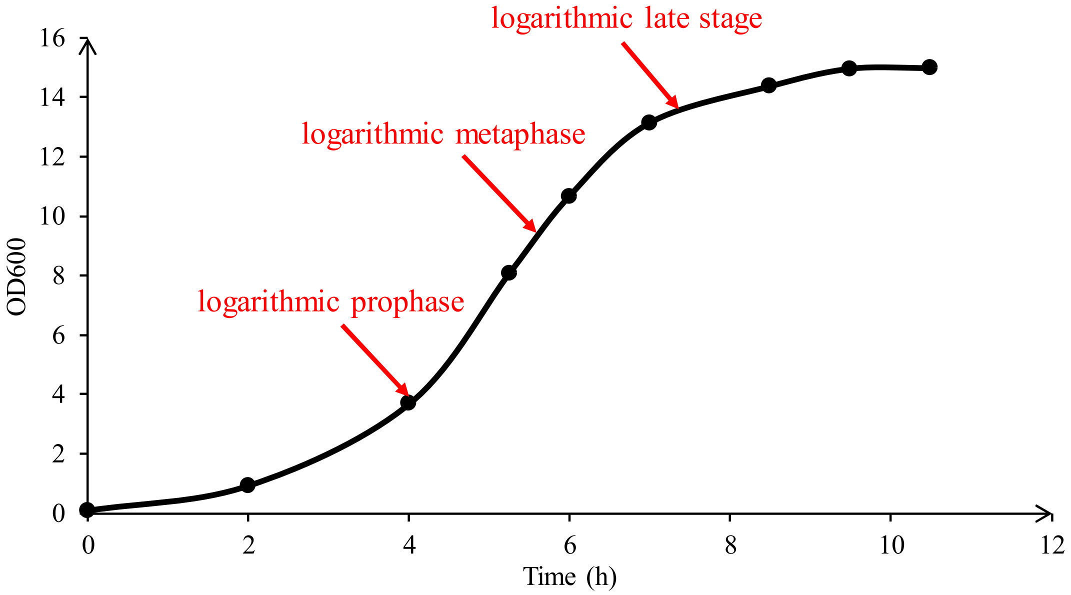

Fig. S1 Growth curve of *E. coli* BL-pα1β2 strain in shake flask

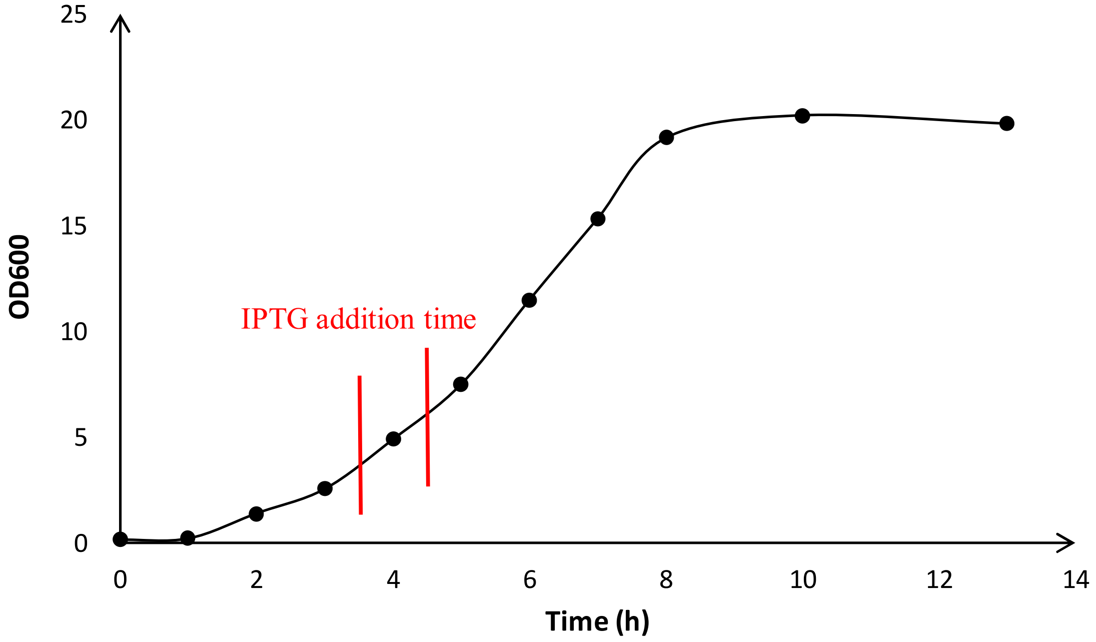

Fig. S2 Growth curve of *E. coli* BL-pα1β2 strain in 5 L-stirred tank fermenter

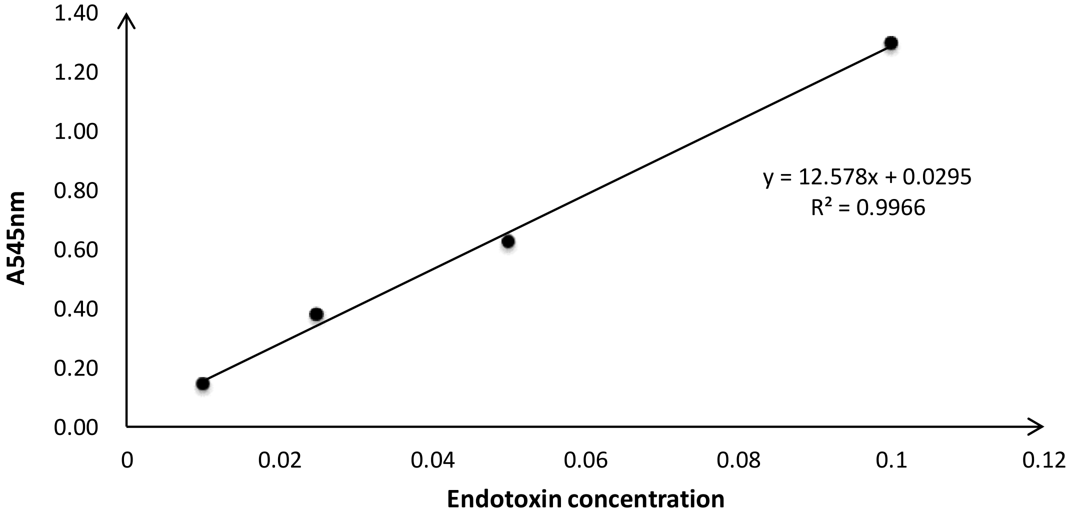

Fig. S3 Standard curve of endotoxin

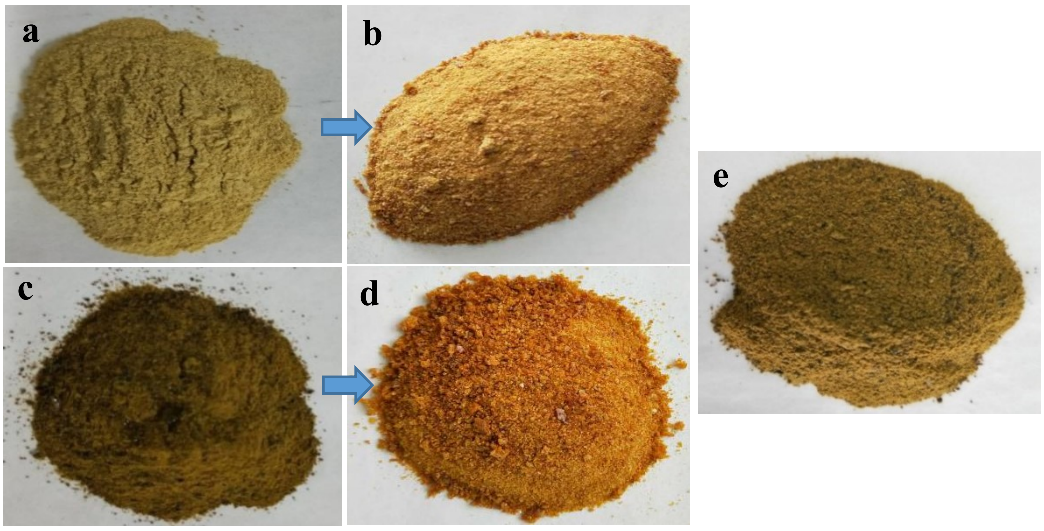

Fig. S4 The morphology of chicken bile powder and the corresponding products

a. Refined chicken bile powder (RCBP). b. Products from RCBP. c. Crude chicken bile powder. d. Products from crude chicken bile powder. e. Natural bear bile powder.

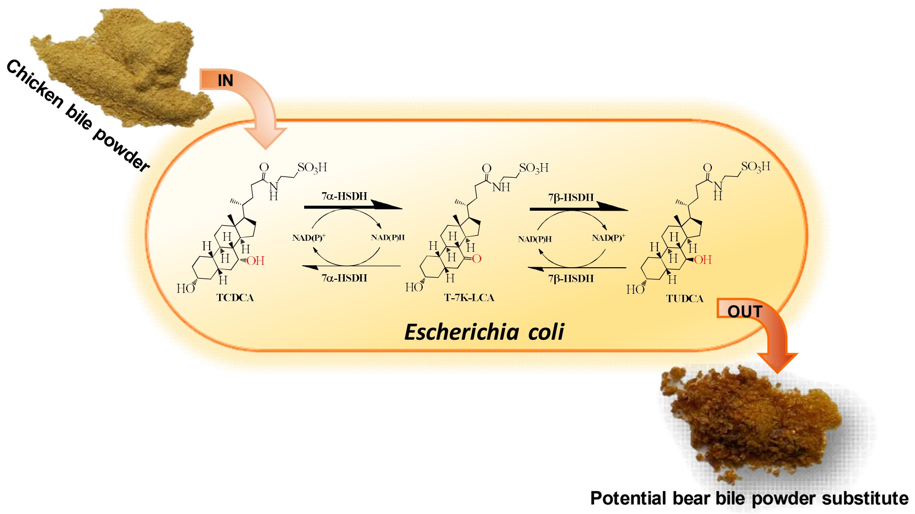

Fig. S5 Engineered *E. coli* factory for potential substitute of bear bile powder
